# Adolescent loneliness and social isolation as predictors of 24-h movement guidelines adherence into adulthood: a prospective study

**DOI:** 10.1186/s13034-025-01020-1

**Published:** 2026-01-05

**Authors:** Yasmin Ezzatvar, Jacinto Muñoz-Pardeza, Rodrigo Yáñez-Sepúlveda, Juan Hurtado-Almonacid, Ignacio Hormazábal-Aguayo, Óscar Martínez-de-Quel, Antonio García-Hermoso

**Affiliations:** 1 Vicerrectoría de Investigación y Postgrado, Universidad de Los Lagos, Osorno, Chile; 2 Lifestyle factors with Impact on Ageing and overall Health (LAH) Research Group, Department of Nursing, University of València, Valencia, Spain; 3 Navarrabiomed, Hospital Universitario de Navarra, Universidad Pública de Navarra (UPNA), IdiSNA, Pamplona, Spain; 4 Faculty of Education and Social Sciences, Universidad Andres Bello, Viña del Mar, Chile; 5 School of Medicine, Espíritu Santo University (Universidad Espíritu Santo, UEES), Samborondón, Ecuador; 6 eFidac Grupo de Investigación. Escuela de Educación Física, Pontificia Universidad Católica de Valparaíso, Valparaíso, Chile; 7 Vicerrectoría de Investigación y Postgrado, Universidad de La Serena, La Serena, Chile; 8 Department of Health Sciences, Public University of Navarre, Tudela, Spain

**Keywords:** Social behavior, Social support, Physical activity, Screen time, Sleep, Adolescence, Young Adult

## Abstract

**Background:**

Loneliness and social isolation are psychosocial factors linked to adverse health outcomes in adolescence, but their associations with the integrated 24-h movement guidelines, covering physical activity, screen time, and sleep, remain poorly understood, particularly over the life course. The aim of the study was to examine the associations of loneliness and social isolation with adherence to the 24-h movement guidelines during adolescence and in sustained patterns from adolescence into adulthood over a 22–24-year follow-up.

**Methods:**

We analyzed longitudinal data from individuals who participated in Waves I (1994–1995, n = 20,603) and V (2016–2018; n = 10,979) of the Add Health study. Loneliness (single CES-D item) and social isolation (frequency of peer interactions) were assessed in adolescence (ages 12–17). Adherence to movement guidelines was self-reported at both waves. Generalized linear models with Poisson regression estimated relative risks (RR) for cross-sectional and sustained (adolescence-to-adulthood) adherence.

**Results:**

At baseline, loneliness was reported by 8.4% and social isolation by 9.4% of adolescents. In women, loneliness was associated with lower adherence to physical activity (RR = 0.87; 95%CI 0.77–0.99), sleep (RR = 0.86; 95%CI 0.79–0.94), and all 24-h movement guidelines (RR = 0.68; 95%CI 0.51–0.90), with associations for sleep (RR = 0.77; 95%CI 0.65–0.91) and all guidelines (RR = 0.37; 95%CI 0.10–0.91) persisting into adulthood. In men, loneliness was associated with lower adherence to sleep (RR = 0.87; 95%CI 0.78–0.97) and all guidelines (RR = 0.78; 95%CI 0.59–0.92), with similar associations observed longitudinally. Social isolation was strongly associated with lower physical activity in both sexes (women: RR = 0.59; 95%CI 0.46–0.75; men: RR = 0.48; 95%CI 0.38–0.61) and with adherence to all guidelines (women: RR = 0.61; 95%CI 0.43–0.87; men: RR = 0.69; 95%CI 0.51–0.93), both cross-sectionally and longitudinally.

**Conclusions:**

Addressing loneliness and social isolation as distinct, yet complementary, correlates of movement behaviors may enhance the effectiveness of strategies aimed at promoting healthier movement patterns and supporting social connectedness.

## Background

Adolescence is a critical period for the development of physical, emotional, and social health [1]. During this stage, maintaining healthy lifestyle behaviors is essential for current and long-term well-being [2]. The physical activity, sleep time and sedentary behaviors are jointly addressed in the 24-h movement guidelines, which emphasize the importance of a balanced distribution of daily time across them to optimize health outcomes [3]. However, adherence to these guidelines remains low among adolescents [4].

Increasing attention has also been paid to the psychosocial dimensions of adolescent health, particularly loneliness and social isolation [5]. Loneliness, defined as the subjective feeling of being alone or disconnected, has emerged as a significant public health concern, especially among young people, as highlighted in the recent report of the WHO Commission on Social Connection [6]. Social isolation, by contrast, refers to an objective lack of social contacts or interactions [7]. Although conceptually distinct, both loneliness and social isolation have been consistently associated with negative health and developmental outcomes during adolescence, including higher levels of depressive and anxiety symptoms, increased risk of self-harm, poorer sleep, lower academic achievement, and greater likelihood of engaging in health-risk behaviors, with potential long-term implications for well-being [8, 9].

Emerging research suggests that loneliness and social isolation may also influence health behaviors, including physical activity [10, 11], sleep [12, 13], and sedentary behavior [11, 12, 14]. Adolescents who feel disconnected from peers may be less likely to participate in physical or social activities and more likely to engage in solitary, sedentary behaviors, such as excessive screen use [15]. Recent evidence from a large cross-sectional study of Chinese adolescents showed that meeting single and combined 24-h movement guidelines was associated with a lower risk of depression, anxiety, and loneliness, with a clear dose–response pattern [16]. However, evidence remains scarce regarding how these psychosocial factors relate to adherence to the integrated 24-h movement guidelines; to our knowledge, only two studies have specifically explored these associations in adolescents [12, 16], and one of them relied on data collected during exceptional contexts such as the COVID-19 pandemic [12], limiting generalizability, particularly in longitudinal frameworks extending into adulthood.

To address this gap, the present study aimed to explore the association between loneliness and social isolation and the likelihood of meeting the 24-h movement guidelines in adolescence and adulthood. Using data from a large, nationally representative cohort, we examined both cross-sectional associations during adolescence and longitudinal patterns of sustained adherence over 22–24-year follow-up.

## Methods

### Population sample and study design

Add Health was originally designed to be nationally representative of U.S. adolescents in grades 7–12 at Wave I (12–17 years old) [17]. Our analytic sample at baseline (n = 20,575) retained this representativeness because all adolescents with available information on loneliness and social isolation were included. The subsample followed into Wave V (37–39 years old) (n = 10,979) reflects expected longitudinal attrition; missingness was primarily due to the absence of information on 24-h movement behaviors rather than selective exclusion. Previous methodological reports from Add Health indicate that the retained cohort remains socio-demographically comparable to the original sample, and no evidence suggests differential attrition associated with loneliness or social isolation. Therefore, although attrition reduced the sample size, the subsample analyzed in this study continues to reflect the socio-demographic diversity of the original nationally representative cohort. Sample sizes shown in Table 1 correspond to participants with complete data for the variables included in the descriptive analyses, which may explain minor discrepancies with the overall numbers reported above.

**Table 1:** Demographic characteristics of the participating subjects by sex at Wave I and V

Wave I	Men (n = 10,180)	Women (n = 10,395)	*p*
Age Wave I, years	16.22 (1.77)	16.07 (1.75)	< 0.001
Race/ethnicity, %			0.001
White	67.3	66.1	
Black or African American	22.7	24.7	
American Indian or Alaska Native	2.0	2.0	
Asian	8.1	7.2	
24-h movement guidelines			
Meets physical activity, %	40.6	18.1	< 0.001
Meets screen time, %	35.7	46.7	< 0.001
Meets sleep time, %	55.6	51.8	< 0.001
Meets all 24-h movement guidelines, %	8.4	4.6	< 0.001
Loneliness, %	6.4	10.3	< 0.001
Never or rarely, %	68.8	57.2	
Sometimes, %	24.8	32.5	
A lot of the time, %	4.8	7.4	
Most of the time or all of the time, %	1.6	2.8	
Social isolation (not at all)*, %	8.0	10.8	< 0.001
1 or 2 times, %	22.6	24.7	
3 or 4 times, %	27.3	26.1	
5 or more times, %	42.1	38.4	

Wave V	Men (n = 5269)	Women (n = 6889)	
Age Wave V, years	38.15 (1.91)	37.88 (1.89)	< 0.001
24-h movement guidelines			
Meets physical activity, %	24.3	20.0	< 0.001
Meets screen time, %	81.5	85.1	< 0.001
Meets sleep time, %	55.4	57.9	0.006
Meets all 24-h movement guidelines, %	12.3	11.0	0.039
Across both waves			
Meets physical activity, %	11.4	4.8	< 0.001
Meets screen time, %	31.1	42.7	< 0.001
Meets sleep time, %	32.7	32.3	0.726
Meets all 24-h movement guidelines, %	1.6	0.8	< 0.001
Depression, %	17.3	30.1	< 0.001

^*^ During the past week, how many times did you just hang out or talk with friends?

The Add Health study was approved by the Institutional Review Board (IRB) at the University of North Carolina at Chapel Hill. Permission to conduct secondary analyses was obtained by the Ethics Committee of the University Hospital of Navarra (PI_2020/143).

### Loneliness

Loneliness was assessed in Wave I using a single item from the 20-item Center for Epidemiological Studies Depression Scale (CES-D) [18], which asked: “How often was the following true during the past week? You felt lonely.” Response options included: “Never or rarely,” “Sometimes,” “A lot of the time,” “Most of the time or all of the time,” “Refused,” and “Don’t know.” Following prior research [19], we created a binary loneliness variable: participants reporting “A lot of the time” or “Most of the time or all of the time” were classified as experiencing high loneliness, while those answering “Never or rarely” or “Sometimes” were categorized as low loneliness. Responses of “Refused” and “Don’t know” were treated as missing.

### Social isolation

Social isolation was measured with the item: “During the past week, how many times did you just hang out or talk with friends?” Response options included: “Not at all,” “1 or 2 times,” “3 or 4 times,” and “5 or more times.” Following a frequency-based approach, we treated this as an indicator of social isolation, where lower frequency responses reflect greater isolation. For descriptive analyses, we retained the four original categories, but for regression models, social isolation was dichotomized: participants who responded “Not at all” were classified as socially isolated with regards to peers, while all others were considered socially connected.

### 24-h movement guidelines

The Canadian 24-h movement guidelines for youth [3] and adults [20] recommend adherence to all three components previously mentioned, with compliance coded as 1 (i.e., meeting the 24-h movement guidelines). In the Add Health surveys, these guidelines were assessed using a standard self-reported recall method, comparable to those employed and validated in prior large-scale epidemiological studies [21, 22].

#### Physical activity

At Wave I, adolescents self-reported past-week moderate-to-vigorous physical activity (MVPA) using three items: “During the past week, how many times did you go rollerblading, roller-skating, skateboarding, or bicycling?”, “During the past week, how many times did you play an active sport, such as baseball, softball, basketball, soccer, swimming, or football?”, and “During the past week, how many times did you exercise, such as jogging, walking, karate, jumping rope, gymnastics or dancing?”. Response options ranged from “none” to “5 or more times” and were scored as 0, 1.5, 3.5, and 6 times, respectively. Since no specific measure assessed guideline adherence, “ ≥ 5 MVPA sessions/week” was used as a proxy, following previous research [23, 24].

At Wave V, MVPA in the past 7 days was assessed using five items: “In the past 7 days, how many times did you bicycle, skateboard, dance, hike, hunt, or do yard work?”, “In the past 7 days, how many times did you roller blade, roller skate, downhill ski, snowboard, play racquet sports, or do aerobics?”, “In the past 7 days, how many times did you participate in gymnastics, weight lifting, or strength training?”, “In the past 7 days, how many times did you participate in individual sports such as running, wrestling, swimming, cross-country skiing, cycle racing, martial arts, or in strenuous team sports such as football, soccer, basketball, lacrosse, rugby, field hockey, or ice hockey?”, and “In the past 7 days, how many times did you play golf, go fishing or bowling, or play softball or baseball?”. Participants reporting five or more instances of these activities were classified as physically active; all others were considered inactive [23]. Finally, participants were classified as having an “active lifestyle” if they met the physical activity recommendations in both adolescence (Wave I) and adulthood (Wave V).

#### Screen time

Screen time was assessed using a previously established scale [23] that included three items: weekly hours spent watching television, watching videos, and playing video or computer games. The responses to these items were summed to calculate total weekly recreational screen time. Compliance with screen time guidelines was defined as reporting no more than 2 h per day at Wave I (adolescence) [3] and no more than 3 h per day at Wave V (adulthood) [20].

#### Sleep time

At both Wave I and Wave V, participants reported their typical daily sleep duration (in hours) in response to the question: “How many hours of sleep do you usually get per day and/or night?” Sleep guideline adherence was determined based on the National Sleep Foundation’s age-specific recommendations: 9–11 h for adolescents aged 12–13, and 8–10 h for those aged 14–17 [25]. For adults in Wave V, meeting sleep duration guidelines was defined as reporting between 7 and 9 h of sleep per day [20].

#### Trajectories of meeting the 24-h movement guidelines

We examined sustained adherence by identifying participants who consistently met each individual movement behavior guideline, physical activity, screen time, and sleep duration, at both Wave I and Wave V. Additionally, a composite indicator of sustained adherence was created as a binary variable, coded as 1 if the participant met all three 24-h movement guidelines at both Wave I and Wave V, and 0 otherwise.

#### Covariates

Sociodemographic information, including age, sex, and race/ethnicity, was collected via in-home interviews. Race/ethnicity was categorized into four groups: White, Black or African American, American Indian or Alaska Native, and Asian. Additionally, a binary variable was included based on the question: “Has a doctor, nurse, or other health care provider ever told you that you have or had depression?”, indicating whether a health professional had ever diagnosed the participant with depression.

#### Statistical analysis

All regression analyses were stratified by sex. Sex differences in descriptive characteristics were examined using *t*-tests for continuous variables and χ^2^ tests for categorical variables. We fitted generalized linear models (GLMs) using Poisson regression with a log link function and robust standard errors to estimate relative risks (RRs) and their 95% confidence intervals (CIs) for each movement behavior outcome, physical activity, screen time, sleep duration, and adherence to the full 24-h movement guidelines, measured at Wave 1 and sustained across both waves.

Analyses were structured in two parts: (1) associations between loneliness, social isolation, and movement-related behaviors during adolescence (Wave I); and (2) associations with sustained adherence to 24-h movement guidelines from adolescence into adulthood. The primary exposure variables were loneliness and social isolation, both assessed at Wave 1. All models were adjusted for age, sex, and race. In analyses focusing on Wave V and sustained adherence, we additionally adjusted for age at Wave 5 and whether the participant had ever been diagnosed with depression.

All analyses were conducted in R (version 4.4.1; R Core Team, Vienna, Austria) using RStudio (version 2024.09.1; RStudio, Boston, MA, USA). Statistical significance was set at a two-tailed *p*-value < 0.05.

## Results

Table 1 presents the demographic characteristics and the proportion of boys and girls meeting the 24-h movement guidelines at Wave I, at Wave V, and across both waves, along with sex differences. At Wave I, boys were more likely to meet physical activity recommendations than girls (40.6% vs. 18.1%, *p* < 0.001), whereas girls were more likely to meet screen time guidelines (46.7% vs. 35.7%, *p* < 0.001). Boys also showed slightly higher adherence to sleep recommendations than girls (55.6% vs. 51.8%, *p* < 0.001). Only a small percentage met all three guidelines, with boys showing a higher prevalence than girls (8.4% vs. 4.6%, *p* < 0.001). Regarding psychosocial variables, loneliness was reported by 6.4% of boys and 10.3% of girls (*p* < 0.001), while social isolation affected 8.0% of boys and 10.8% of girls (*p* < 0.001).

When examining sustained adherence across both waves, compliance was notably low. Girls showed lower sustained physical activity (4.8% vs. 11.4%, *p* < 0.001) but higher screen time adherence (42.7% vs. 31.1%, *p* < 0.001). Fewer than 2% of adolescents met all three recommendations over time, with boys more likely to do so than girls (*p* < 0.001). Women also reported significantly higher rates of depression diagnosis compared to men (30.1% vs. 17.3%, *p* < 0.001).

**Fig. 1:**
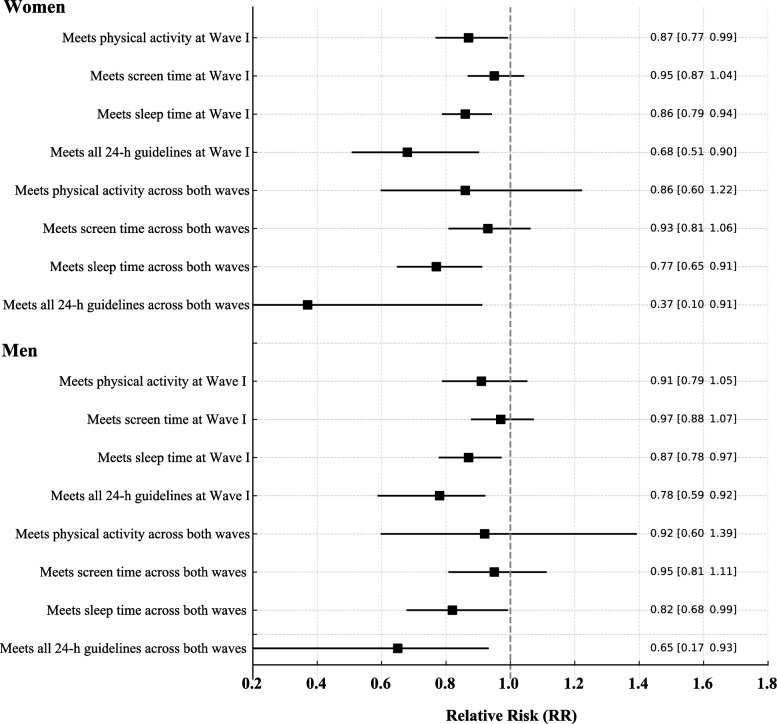
Relative risks for the association between loneliness and adherence to movement behavior guidelines at wave 1 and sustained across waves 1 and 5

Figure 1 displays the RR and 95% CI for the association between loneliness and movement behaviors. Among women, loneliness at Wave I was significantly associated with a lower likelihood of meeting recommendations for physical activity (RR = 0.87; 95% CI 0.77–0.99), sleep (RR = 0.86; 95% CI 0.79–0.94), and the combined 24-h movement guidelines (RR = 0.68; 95% CI 0.51–0.90), with no significant association found for screen time. These associations persisted over time, with loneliness at Wave I predicting lower sustained adherence to sleep (RR = 0.77; 95% CI 0.65–0.91) and to the full 24-h movement pattern (RR = 0.37; 95% CI 0.10–0.91) across Waves I and V. Among men, loneliness at Wave 1 was linked to reduced compliance with sleep (RR = 0.87; 95% CI 0.78–0.97) and 24-h movement guidelines (RR = 0.78; 95% CI 0.59–0.92). Similar associations were observed at follow-up for sleep (RR = 0.82; 95% CI 0.68–0.99) and 24-h behaviors (RR = 0.65; 95% CI 0.17–0.93). No significant associations were found for physical activity or screen time in men.

**Fig. 2:**
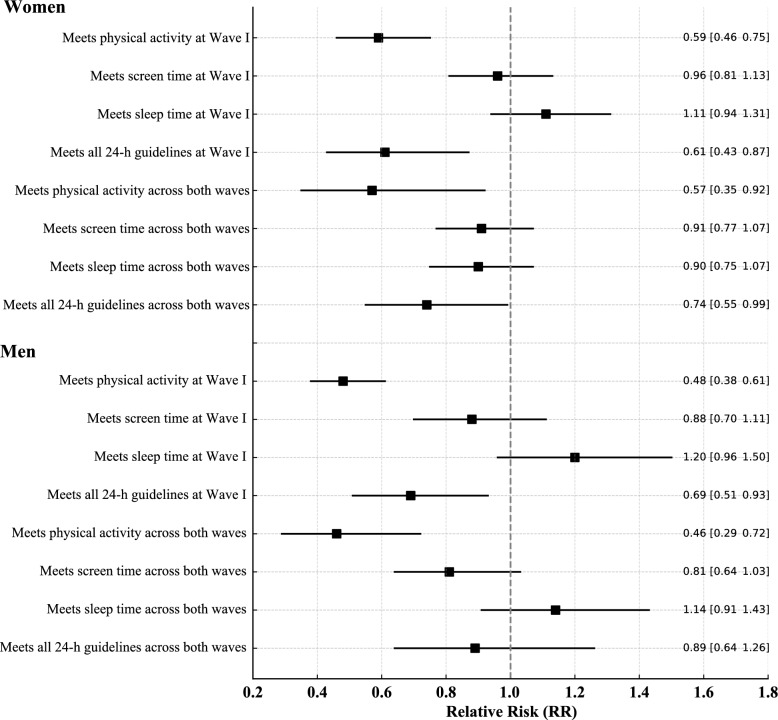
Relative risks for the association between social isolation and adherence to movement behavior guidelines at wave 1 and sustained across waves 1 and 5

Social isolation at Wave I among women was significantly associated with a lower likelihood of meeting physical activity recommendations (RR = 0.59; 95% CI 0.46–0.75) and adhering to all 24-h movement guidelines (RR = 0.61; 95% CI 0.43–0.87). No significant associations were observed for screen time or sleep duration. These findings remained at follow-up, where social isolation continued to predict lower compliance with physical activity (RR = 0.57; 95% CI 0.35–0.92) and 24-h guidelines (RR = 0.74; 95% CI 0.55–0.99). Among men, social isolation at Wave I was linked to lower adherence to physical activity (RR = 0.48; 95% CI 0.38–0.61) and 24-h guidelines (RR = 0.69; 95% CI 0.51–0.93). At follow-up, it remained significantly associated with physical activity (RR = 0.46; 95% CI 0.29–0.72), with no significant associations found for sleep or screen time (Fig. 2).

## Discussion

Our study provides novel evidence that both loneliness and social isolation experienced during adolescence are significantly associated with lower adherence to the 24-h movement guidelines, particularly for physical activity, sleep and all three movement behaviors. Especially, these associations were more pronounced and consistent among women, and many persisted when considering both waves jointly, suggesting that the effects of early social disconnection may extend beyond adolescence and potentially have lasting behavioral health consequences.

### Association between loneliness and 24-h movement behaviors

Our findings indicate that experiencing loneliness during adolescence was associated with lower adherence to physical activity recommendations among women. This pattern is consistent with evidence from a large UK cohort of young adults aged 18 years, which showed that loneliness was associated with less frequent physical activity, poorer sleep, and more somatic symptoms, even after adjusting for depression, suggesting an independent link that may operate in a reinforcing cycle [26]. Collectively, this evidence supports the notion that perceived social disconnection can diminish both the motivation and the opportunities for participating in active, socially oriented behaviors [27]. One possible explanation for the sex-specific association is that adolescent women are more likely to engage in physical activities with a strong social component, such as group exercise or team sports, and tend to place greater value on peer approval and connectedness in these contexts [28]. Consequently, the absence of supportive social ties may disproportionately impact their participation compared to men, who may be more engaged in individual or less socially dependent activities [29].

The association with sleep, observed in both sexes, may reflect the link between loneliness and dysregulated sleep patterns [30], potentially mediated by psychological distress, heightened physiological arousal (e.g., elevated hypothalamic–pituitary–adrenal axis activity and cortisol), and increased vigilance for threat [31, 32]. The persistence of these associations into adulthood suggests that loneliness experienced early in life may set long-term behavioral trajectories. This is particularly relevant given that only few studies to date have examined loneliness in relation to all three 24-h movement behaviors in combination, for example, Hua et al. [16] recently reported cross-sectional associations between adherence to the 24-h movement guidelines and loneliness among Chinese adolescents. Our longitudinal approach adds to this evidence by investigating how loneliness in adolescence relates to subsequent adherence to integrated movement behavior patterns over time. In addition, our results align in part with findings from Tandon et al. [12], who examined loneliness during the COVID-19 pandemic and its association with adherence to 24-h movement behaviors among more than 40,000 Canadian adolescents. Their cross-sectional analyses showed that increased loneliness was associated with insufficient sleep and higher screen time, although associations with physical activity were weak or clinically negligible. While their study was conducted under exceptional circumstances and cannot address long-term behavioral patterns, it nonetheless supports the notion that loneliness relates to less healthy movement behavior profiles in adolescence. Our longitudinal evidence expands on this and demonstrates that loneliness experienced during adolescence is associated with reduced adherence at that developmental stage and predicts poorer movement behavior trajectories extending into adulthood.

Interestingly, we did not observe significant associations with screen time, which could indicate that screen-based sedentary activities may serve as a coping mechanism for lonely adolescents rather than a behavior directly reduced by social connection [33]. It is worth noting, however, that the present study was conducted in 1994–1995 and 2016–2018, when the nature, prevalence, and context of screen use differed from current patterns, which may in turn influence its behavioral correlates and health impacts. However, another more recent study from Canada followed more than 20,000 secondary school students for one year and found that, while loneliness and screen time (television, video games, internet, text messaging) were correlated cross-sectionally, neither predicted the other over time [14]. Similarly, a three-year longitudinal study reported no evidence that baseline screen time predicted later loneliness, or that loneliness predicted subsequent increases in screen time, despite cross-sectional associations being present at each wave [34]. In other words, loneliness did not lead to increased screen time, nor did screen time lead to greater loneliness over the course of the study, suggesting that the relationship between these variables in adolescents may be more complex and context-dependent than previously assumed [35].

### Association between social isolation and 24-h movement behaviors

Social isolation was also strongly linked to lower adherence to physical activity and the integrated 24-h movement guidelines, in both adolescence and adulthood. Our findings confirm a multi-country cross-sectional analysis of data from 79 countries from the Global School-Based Student Health Survey, which showed that adolescents with fewer friends and lower social participation had markedly lower odds of meeting physical activity recommendations, a pattern consistent across most national contexts [11]. The stronger associations observed for physical activity may be explained by the fact that many opportunities for exercise during adolescence, such as team sports, recreational play, or peer-based activities, are inherently social [28]. Adolescents who are physically active also tend to form friendships with peers who are likewise active [36], and higher-quality friendships have been linked to greater engagement in physical activity [37]. The absence of regular social engagement may therefore reduce exposure to these opportunities and weaken social norms that encourage activity [28, 38].

Longitudinal evidence suggests that stronger peer support during the transition from late adolescence to young adulthood may help promote higher physical activity levels and foster more stable patterns over time [39]. Moreover, profiles of adolescent physical activity that are embedded in social or organized contexts, such as team or group-based activities, are more likely to be maintained into emerging adulthood, compared to individual activity patterns [40].

Social isolation was not associated with screen time in our analyses,, which captures only recreational screen use rather than the broader construct of sedentary behavior [15]. Adolescents engage in various sedentary activities that are not strongly dependent on peer interaction (e.g., schoolwork, studying, commuting), which may weaken observed associations when screen time alone is used as a proxy. Furthermore, screen-based activities can occur in both socially connected and disconnected contexts and may serve as a coping mechanism irrespective of the degree of objective isolation [14]. It is therefore possible that different patterns would emerge if total sedentary behavior, including all low-energy-expenditure sitting or reclining activities, were examined.

The absence of associations between social isolation and sleep may further support this distinction, suggesting that sleep is more sensitive to the subjective experience of loneliness than to the objective frequency of peer contact. Prior research has shown that loneliness is closely linked to heightened vigilance, perceived threat, and cognitive–emotional arousal, all of which interfere with sleep regulation [30, 32]. In contrast, our single-item indicator of social isolation may capture opportunities for social interaction but not these internal processes that are more proximal to sleep disruption. This distinction may explain why loneliness, but not social isolation, predicted lower adherence to sleep recommendations.

The maintenance of the association between adolescent social isolation and poorer adherence to the 24-h movement guidelines into adulthood suggests that early deficits in social connectedness can have lasting behavioral consequences. Social isolation in adolescence may limit opportunities for engaging in team sports, recreational play, and other peer-based activities, thereby reducing physical activity and weakening social norms that discourage excessive sedentary time [11]. Supportive peer networks can enhance self-efficacy [41], strengthen activity-related habits [42], and reinforce social norms that value balanced movement behaviors [43], while also providing access to environments conducive to an active lifestyle [44]. These combined effects may help explain why adolescents with richer social connections are more likely to maintain adherence to the integrated 24-h movement guidelines into adulthood.

The distinction between objective social isolation and subjective loneliness has important implications for prevention in educational and community settings [27]. Interventions addressing loneliness may need to prioritize emotional support, perceived belonging, and skills for managing social-emotional processes. In contrast, initiatives targeting social isolation should focus on increasing opportunities for meaningful peer interaction, participation in group activities, and expanding adolescents’ social networks through schools and community programs. Future efforts could also include policy-level strategies that promote social connection, such as school-based social connection curricula, community clubs, or digital-age interventions designed to foster healthy online interactions [6].

### Strength and limitations

A key strength of this study is the use of a large, nationally representative sample with over two decades of follow-up, enabling the examination of both cross-sectional and sustained associations from adolescence into adulthood. Nonetheless, certain limitations should be considered. The operationalization of social isolation relied solely on the frequency of informal peer interactions, which, although pragmatic, may not capture its broader structural (e.g., network size, diversity of roles) and functional (e.g., perceived support, reciprocity) dimensions [45]. This approach may underestimate disconnection from other social contexts or overlook qualitative aspects of relationships, potentially attenuating observed associations. Moreover, this single-item indicator has not been formally validated as a comprehensive measure of social isolation and focuses exclusively on peer interactions, not on family or other social ties. The use of self-reported measures also may introduce bias, and the physical activity items did not capture information on duration or habitual patterns. We inferred activity intensity and duration by applying “five or more MVPA sessions per week” as a proxy for meeting physical activity recommendations. Furthermore, physical activity was assessed using frequency-based self-report items, and our proxy of ≥ 5 MVPA sessions per week does not capture session duration or intensity in detail, thereby limiting the precision with which adherence to time-based physical activity recommendations can be determined. Moreover, physical activity items were not identical across Waves I and V, which may partly account for the observed difference in the proportion of boys/men meeting ≥ 5 MVPA sessions/week across waves. In addition, data from 1994–1995 were used to evaluate adherence to guidelines that were established later. Over the past two decades, screen use has shifted markedly, from traditional television viewing to streaming and other digital formats, contributing to overall increases in screen time. It is therefore possible that associations between loneliness, social isolation and screen-based behaviors would differ if assessed in the contemporary digital environment, where screen use is more ubiquitous, multifunctional, and socially embedded. Finally, the sedentary behavior component was operationalized using self-reported recreational screen time only. This indicator does not capture other important domains of sedentary behavior (e.g., sitting at school, work, or during transport) and some screen-based activities can occur while being physically active. It should also be acknowledged that all 24-h movement behaviors were assessed using self-reports; although appropriate for large-scale cohorts initiated in the 1990s, device-based methods such as accelerometers now provide continuous and more accurate assessment of physical activity, sedentary behavior, and sleep, and their absence may contribute to measurement error.

## Conclusions

While both loneliness and social isolation were associated with lower adherence to the combined 24-h movement guidelines, as well as with specific individual behaviors, their behavioral correlates and persistence patterns differed. Loneliness, reflecting the subjective perception of social disconnection, was associated with both physical activity and sleep duration in women, and with physical activity in men. In contrast, social isolation, representing an objective lack of social contact, was consistently and strongly associated with physical activity but showed no significant relationship with sleep [46].

These distinctions highlight the importance of addressing both constructs independently in public health strategies, as interventions fostering perceived social support may influence a broader range of movement behaviors, whereas those enhancing objective social engagement may be particularly critical for sustaining active lifestyles.

## Data Availability

Due to our data protection agreements with the participating cohort study, we are unable to share individual-level data with third parties. According to Add Health’s data access policy, researchers can submit data requests to the steering committee. These requests will be reviewed promptly for confidentiality, data protection, and intellectual property considerations, and will not be unreasonably denied. Researchers registered with Add Health can apply for access to its database by submitting an application ( https://data.cpc.unc.edu/projects/2/view).
